# Clusters in the Spread of the COVID-19 Pandemic: Evidence From the G20 Countries

**DOI:** 10.3389/fpubh.2020.628789

**Published:** 2021-01-18

**Authors:** Tian Meng

**Affiliations:** Shanghai Sci-Tech Finance Institute, Shanghai University, Shanghai, China

**Keywords:** the COVID-19 pandemic, the COVID-19 cases, the COVID-19 deaths, convergence clustering test procedure, the G20

## Abstract

This study tests the validity of the club convergence clustering hypothesis in the G20 countries using four measures of the spread of the COVID-19 pandemic: total number of confirmed cases per million people, new cases per million people, total deaths per million people, and new deaths per million people. The empirical analysis is based on the daily data from March 1, 2020, to October 10, 2020. The results indicate three clusters for the per capita income, two clusters for total cases per million people, and new cases per million people. Besides, there are only one and two clusters for total deaths per million people and new deaths per million people. Potential policy implications are also discussed in detail.

## Introduction

In this paper, we examine the validity of the club convergence clustering hypothesis in the G20 countries using four indicators of the spread of the COVID-19 pandemic: total number of confirmed cases per million people, new confirmed cases per million people, total deaths per million people, and new deaths per million people. It is essential to examine the validity of the convergence clustering hypothesis in the G20 countries related to the indicators of the spread of the COVID-19 pandemic. Indeed, whether there are significant clusters in the spread of the COVID-19 pandemic can be particularly important for policy implications, such as lockdowns and limitations on business and social life. COVID-19 pandemic significantly affects every aspect of the global economy (1, 2). Therefore, forecasting the COVID-19 pattern in different countries is significant.

There are previous papers to analyze the spread of the pattern of the COVID-19 pandemic. For example, Katul et al. (3) show a significant global convergence in the generic spread mechanisms of the COVID-19. However, the authors focus on the data until early 2020. Kuniya (4) also examines the impact of an emergency state for the first wave of the COVID-19 in Japan for the period from April 7 to May 25, 2020. The author finds that the state of emergency has provided to 80% decline in the contact rate. Therefore, there is a significant convergence in the spread of the COVID-19 pandemic in Japan during concern. Chimmula and Zhang (5) forecast the infectious diseases related to the COVID-19 outbreak in Canada. The authors show that the spread of the COVID-19 pandemic in Canada follows a stationary forecasting process. Shabani and Shahnazi (6) considered the data of the COVID-19 cases from February 9, 2020, to July 27, 2020, to analyze COVID-19's spatial distribution dynamics. For this purpose, the authors applied the Markov Chain, while also used the Spatial Markov Chain. The findings indicate that the COVID-19 in 40 Asian countries have a unit root characteristics with the domestic policies. Besides, the neighboring countries have significant effects on the spread of COVID-19. Ismail et al. (7) confirm the evidence of convergence for the indicators on the spread of COVID-19 in 187 countries.

This study follows the current developments in the literature. It aims to examine the validity of the club convergence clustering hypothesis in the G20 countries using four indicators of the spread of the COVID-19 pandemic: total cases per million people, new cases per million people, total deaths per million people, and new deaths per million people. We use the daily data from March 1, 2020, to October 10, 2020.

A thorough search of the relevant literature yielded only one related article. This is the first study to use the club convergence clustering method to examine the spread of the COVID-19 pandemic in different countries. The results indicate two clusters for the per capita income, three clusters for total cases per million people, and new cases per million people. Besides, there are only one and two clusters for total deaths per million people and new deaths per million people. These findings suggest some substantial implications in the G20 countries. For example, the policymakers in these should implement measures for controlling the spread of the COVID-19 pandemic, and some countries have different dynamics in the spread of the COVID-19 pandemic. This main evidence should be some significant policy implications for these countries since the risks related to the COVID-19 significantly greater in some countries than others. Furthermore, emerging countries are seemed to be heavily affected by the COVID-19 pandemic.

The remaining parts of the study are structured as follows: Section Data and Club Convergence Methodology provides the details of the data and the club convergence methodology. The empirical results are stated in Empirical Findings. Section Conclusion concludes the study with possible implications of the findings.

## Data and Club Convergence Methodology

### Data

We examine possible cluster and club convergence dynamics for four indicators of the spread of the COVID-19 pandemic: total cases per million people, new cases per million people, total deaths per million people, and new deaths per million people. The empirical analysis is based on the daily data for the period from March 1, 2020, to October 10, 2020, in the G20 countries (19 countries excluding the European Union): Argentina, Australia, Brazil, Canada, China PR, France, Germany, India, Indonesia, Italy, Japan, Mexico, the Russian Federation, Saudi Arabia, South Africa, South Korea, Turkey, the United Kingdom, and the United States. The list of countries, including the country id of the countries in the empirical analyses, are provided in Table 1. The frequency of the panel data is daily. The data are downloaded from the dataset of Hasell et al. (8), so-called the *Data on COVID-19 (Coronavirus) by Our World in Data* project (https://github.com/owid/covid-19-data/tree/master/public/data).

**Table 1:** Countries in the dataset.

**ISO Code**	**Country**	**Country ID**
ARG	Argentina	1
AUS	Australia	2
BRA	Brazil	3
CAN	Canada	4
CHN	China, PR	5
FRA	France	6
DEU	Germany	7
IND	India	8
IDN	Indonesia	9
ITA	Italy	10
JPN	Japan	11
MEX	Mexico	12
RUS	Russian Federation	13
SAU	Saudi Arabia	14
ZAF	South Africa	15
KOR	South Korea	16
TUR	Turkey	17
GBR	The United Kingdom	18
USA	The United States	19

Descriptive statistics of four indicators of the spread of the COVID-19 pandemic: total cases per million people, new cases per million people, total deaths per million people, and new deaths per million people are reported in Table 2.

**Table 2:** Descriptive statistics.

**Indicator**	**Total Cases per Million People**	**New Cases per Million People**	**Total Deaths per Million People**	**New Deaths per Million People**
Mean	2,858	34.83	144.8	1.287
Max.	23,785	380.8	703.9	74.14
Min.	0.002	0.000	0.000	0.000
Std. Dev.	4,135	53.36	199.6	2.588
Obs.	4,256	4,256	4,256	4,256

### Club Convergence Methodology

Phillips and Sul (9, 10) propose a novel approach for identifying the stochastic properties of convergence and defining different convergence clubs among the panel units over time. The methodology assumes the time-varying model with nonlinear nature, and it offers a mechanism of nonlinear transition. The best way of this approach is that it can also be applied in the panel data with unit root, or it does not assume homogeneous (common) factors in the data-generating algorithm. Besides, Phillips and Sul (9, 10) club convergence methodology captures each country's heterogeneity within the panel dataset. Hence, the club convergence procedure considers the dynamics of the COVID-19 spread among the G20 countries in a panel dataset. The COVID-19 spread rate in each county can be defined by the panel dataset, which may follow different convergence dynamics. Therefore, the club convergence procedure is a suitable test for the convergence dynamics of the COVID-19 spread among the G20 countries. This paper aims to examine the different convergence club features in the COVID-19 spread among the G20 countries. We can define the club convergence procedure as such:

The series X_it_ captures an indicator of the COVID-19 spread for country *i* at time *t*, and i = 1,2,…, N; *t* = 1,2,…,19. At this stage, Phillips and Sul (9, 10) decompose the variable into two components: First is the common component of cross-sectional dependence in a panel dataset, g_it_, and transitory component, a_it_, as such:

(1) $$\begin{array}{l} {\text{X}}_{\text{it}}={\text{g}}_{\text{it}}+{\text{a}}_{\text{it}} \end{array}$$

Phillips and Sul (9, 10) define the Equation (1) as the common and the idiosyncratic components. At this stage, the variable follows nonlinear stochastic properties, as such:

(2) $$\begin{array}{l} {\text{X}}_{\text{it}}=\left(\frac{{\text{g}}_{\text{it}}\;+\;{\text{a}}_{\text{it}}}{{\text{μ}}_{\text{t}}}\right){\text{μ}}_{\text{t}}={\text{δ}}_{\text{it}}{\text{μ}}_{\text{t }}\text{for all i and t}, \end{array}$$

Where, μ_t_ captures the common component and δ_it_ indicates the time-varying idiosyncratic component. δ_it_ denotes the relative difference between common trend component μ_t_ and the value of X_it_ is an indicator of the spread of the COVID-19 in a country *i* at time *t*.

Let us take the deaths from the COVID-19 per million people as an example. μ_t_ denotes a common trend of the COVID-19 per million people in whole 19 countries. δ_it_ captures each country's relative share in terms of the COVID-19 per million people in the common trend in the G20 countries. The baseline approach of club convergence approach of Phillips and Sul (9, 10) is to define the time-varying load δ_it_, and time-varying load will determine the dynamics of the club convergence in terms of the power of convergence. Furthermore, Phillips and Sul (9, 10) calculate a transition coefficient, which can be defined as h_it_. Transition coefficient is based on the time-varying factor loadings (δ_it_), as such:

(3) $$\begin{array}{l} {\text{h}}_{\text{it}}=\frac{{\text{X}}_{\text{it}}}{\frac{1}{\text{N}}\sum_{\text{i}=1}^{\text{N}}{\text{X}}_{\text{it}}}=\frac{{\text{δ}}_{\text{it}}{\text{μ}}_{\text{t}}}{\frac{1}{\text{N}}\sum_{\text{i}=1}^{\text{N}}{\text{δ}}_{\text{it}}{\text{μ}}_{\text{t}}}=\frac{{\text{δ}}_{\text{it}}}{\frac{1}{\text{N}}\sum_{\text{i}=1}^{\text{N}}{\text{δ}}_{\text{it}}} \end{array}$$

In Equation (3), h_it_ indicates a transition term, which measure δ_it_ related to the average of the panel at time *t*. At this stage, the transition term defines a transition nature for source countries *i* relative to the average of the panel dataset of the G20 countries. All indicators used the filter provided by Hodrick and Prescott (11) to remove the cyclical component. Following Ravn and Uhlig (12), lambda is defined 1600 × (365/4)^∧4^ for daily data. The filtered coefficient for transition parameter is represented by $\hat{h}$_*it*_, and an extracted time-trend is defined as ${\hat{X}}_{it}$.

Furthermore, the club convergence test procedure also defines the cross-sectional variance ratio, $\frac{{\text{H}}_{1}}{{\text{H}}_{\text{t}}}$, which can be defined as follows:

(4) $$\begin{array}{l} {\text{H}}_{\text{t}}=\frac{1}{\text{N}}\sum_{\text{i }=\;1}^{\text{N}}{\left({\hat{\text{h}}}_{\text{it}}-1\right)}^{2} \end{array}$$

At this stage, Phillips and Sul (9, 10) show that the transition parameter H_t_ is defined within a limit form, which can be written as such:

(5) $$\begin{array}{l} {\text{H}}_{\text{t}}\sim \frac{A}{L{\left(t\right)}^{2}t^{2\alpha }}\;as\;t\to \text{∞} \end{array}$$

In Equation (5) *A* is a constant term, and *A* > 0, *L(t)* is the function of time, and α indicates the speed of convergence. Phillips and Sul (9, 10) define *log t* regression to test the validity of the null hypothesis of convergence. The null hypothesis can be written as *H*_0_ : δ_*i*_ = δ and α ≥ 0 and against *H*_1_ : δ_*i*_ ≠ δ for all *i* or α < 0.

Furthermore, Phillips and Sul (9, 10) estimate the following Ordinary Least Squares (OLS) equation:

(6) $$\begin{array}{l} \mathrm{Log}\left(\frac{{\text{H}}_{1}}{{\text{H}}_{\text{t}}}\right)-2log\text{L}\left(\text{t}\right)=\hat{\text{a}}+\hat{\text{b}}\log\text{t}+\hat{\text{c}}{\left(\log\text{ t}\right)}^{2}+{\hat{\text{u}}}_{\text{t}} \end{array}$$

In Equation (6), L(t) = log(t + 1), the fitted coefficient of log t is $\hat{\text{b}}=2\hat{\text{α}}$, and $\hat{\text{α}}$ is the estimate of α in the null hypothesis. The authors include the *squares of log t* to enhance the test procedure's power by capturing nonlinearity in the series. The test procedure considers the initial condition by removing a fraction of the sample in the estimated regression. The initial condition requires a starting point *t* = [rT] with r > 0. Phillips and Sul (9, 10) set r = 0.3. The authors estimate the coefficient of $\hat{\text{b}}$ by providing the standard errors in the use of Heteroskedasticity and Autocorrelation Consistent (HAC) of the long-run variance in residuals to perform the one-sided *t*-test of null α ≥ 0. Hence the *t*-test statistic ${\text{t}}_{\hat{\text{b}}}$ is based on the normal distribution, and if ${\text{t}}_{\hat{\text{b}}}$ < –1.645, the null hypothesis of club convergence will be rejected.

Finally, Phillips and Sul (9, 10) discuss that the rejection of the null of club convergence does not mean that there cannot be sub-group convergence in the panel dataset. It is important to note that the club convergence test procedure is defined for detecting cluster units. Using the club convergence test procedure, we examine the club convergence dynamics in the G20 countries over the period under concern. The club convergence is defined as log t regressions with the following main issues:

1) Ordering: Order the *X*_*it*_ series following the last observation in the panel dataset.
2) Group Formation: Calculate t-statistic ${\text{t}}_{\hat{\text{b}}}\left(\text{k}\right)$ for each country (*k*) and select country or countries for the core group.
3) Membership of the Club: Find the country for membership in the core group by including each remaining country separately, following the results of *log* *t* tests. A new county will be added to the club if the calculated t-statistic is higher than zero.
4) Recursion and Stop: Finally, log *t*-tests are applied for the group of unselected countries. If the cluster of countries converges in the first club, a second club will be formed. If there is no club convergence, sub-convergent club clusters will be investigated. If no subgroups are defined for the remaining countries, they will be defined as countries with a divergence pattern.

## Empirical Findings

Table 3 provides the club convergence results for four indicators of the spread of the COVID-19 pandemic: total cases per million people, new cases per million people, total deaths per million people, and new deaths per million people.

**Table 3:** Results of the club convergence tests for indicators of the spread of the COVID-19.

**Indicators of the spread of the COVID-19**	**Clubs**										**$\hat{\text{b}}$**	***t*-statistic**
Total cases per million people	(1)										−0.025	−0.550
	1	3	5	7	8	9	11	12	13	15		
	16	17	19									
	(2)										−0.090	−0.146
	6	14	18									
	(3)										0.417	74.4
	2	4	10									
New cases per million people	(1)										−0.098	−2.011
	1	2	3	4	5	6	7	8	9	10		
	11	12	15	16	17	18	19					
	(2)										−0.820	−0.919
	13	14										
Total deaths per million people	(1)										0.651	179.6
	1	2	3	4	5	6	7	8	9	10		
	11	12	13	14	15	16	17	18	19			
New deaths per million people	(1)										0.031	0.191
	1	2	3	5	8	9	11	12	13	14		
	15	16	17	19								
	(2)										−0.868	−3.689
	4	6	7	10	18							

In terms of the findings of the club convergence test for the total COVID-19 cases per million people, there are three clubs. The log t regression results for the first club consisting of 13 countries with the *t*-statistic of −0.55, and the null hypothesis of convergence can be rejected. The second club consists of three countries (France, Saudi Arabia, and the United Kingdom) with the *t*-statistic −0.146, and the null hypothesis of convergence can be rejected. Finally, the third club shows three countries (Australia, Canada, and Italy) with the t-statistic 74.4, and the null hypothesis of convergence cannot be rejected.

There are two clubs in terms of the club convergence test results for the new COVID-19 cases per million people. The log t regression findings for the first club consisting of 17 countries with the *t*-statistic of −2.011 and the null hypothesis of convergence cannot be rejected. The second club includes two countries (the Russian Federation and Saudi Arabia) with a t-statistic −0.82, and the null hypothesis of convergence can be rejected.

When we look at the club convergence test findings for the total COVID-19 deaths per million people, only one club consists of all countries in the dataset. The log *t* regression results for the only club consisting of all countries with the t-statistic of 179.6 and the null hypothesis of convergence cannot be rejected.

There are two clubs in terms of the club convergence test results for new deaths per million people. The log *t* regression findings for the first club consisting of 14 countries with the *t*-statistic of 0.191 and the null hypothesis of convergence can be rejected. Furthermore, the second club consists of five countries (Canada, France, Germany, Italy, and the United Kingdom) with a t-statistic −3.689, and the null hypothesis of convergence cannot be rejected.

## Conclusion

In this paper, we examined the validity of the club convergence clustering hypothesis in the G20 countries using four indicators of the spread of the COVID-19 pandemic: total cases per million people, new cases per million people, total deaths per million people, and new deaths per million people. We used the daily data from March 1, 2020, to October 10, 2020. We followed the club convergence clustering methodology of Phillips and Sul (9, 10) to model the time-varying nature of the spread of the COVID-19 pandemic and capture different fighting policy pandemic strategies. We observed that the cases and deaths related to the COVID-19 pandemic have a nonlinear nature and converge among the G20 countries.

We observed three clusters for the per capita income and two clusters for total cases per million people and new cases per million people. Besides, there are only one and two clusters for total deaths per million people and new deaths per million people. These results indicate that although policymakers in different countries have different solutions to the total pandemic deaths per million, they have similar stochastic properties in the G20 countries. This evidence can be related to the fact that the treatment of the COVID-19 virus has not been fully provided in the globe and the deaths due to the COVID-19 virus has somehow a random nature. Our results also indicate that if there will be no prevention, the countries with the low-level of COVID-19 spread will converge toward a pandemic's long-run level, which is the United States' case. Different characteristics of the countries have negligible effects on the spread of the COVID-19, particularly when we focus on the club convergence dynamics of the death ratios related to the COVID-19.

In terms of new deaths, Canada, France, Germany, Italy, and the United Kingdom are different countries. The death ratios per million people have decreased in these countries over time, creating a new club for these countries. In terms of other developed and developing G20 countries, there is another club convergence procedure. When we look at the new cases for the COVID-19, only the Russian Federation and Saudi Arabia have a different nature for convergence. Other countries have a similar pattern for the new cases for the COVID-19. The differences between the Russian Federation and Saudi Arabia are related to these countries' leading oil-exporters in the World economy. Note that the oil prices have significantly declined during the COVID-19.

Given that there are autocratic regimes in these countries, they may be underestimating the number of new cases to show the situation better. In terms of total cases for the COVID-19, there are three different clubs, and they are hard to explain. This issue is the limitation of our study. Future papers can focus on more countries to analyze the club convergence clustering hypothesis's validity in larger panel datasets, which should have more countries and higher time dimensions.

## Data Availability Statement

Publicly available datasets were analyzed in this study. This data can be found here: https://github.com/owid/covid-19-data/tree/master/public/data.

## Author Contributions

The author confirms being the sole contributor of this work and has approved it for publication.

## Conflict of Interest

The author declares that the research was conducted in the absence of any commercial or financial relationships that could be construed as a potential conflict of interest.
